# Tumor specific cytokine pattern explored in renal cell carcinoma

**DOI:** 10.1371/journal.pone.0358533

**Published:** 2026-09-29

**Authors:** Xu Chen, Zhuangyao Liao, Rochelle Wickramasekara, Ying Luo, Panning Wang, Sihao Wang, Yunru Chen, Hang Zhou, Shuhong Luo, Shaopeng Qiu, Ruo-Pan Huang

**Affiliations:** 1 Department of Urology, The First Affiliated Hospital, Sun Yat-Sen University, Guangzhou, Guangdong, China; 2 RayBiotech, Inc., Peachtree Corners, GeorgiaUnited States of America; 3 RayBiotech, Inc., Guangzhou, Guangdong, China; 4 Department of Urology, Hui Ya Hospital of the First Affiliated Hospital, Sun Yat-Sen University, Huizhou, Guangdong, China; 5 South China Biochip Research Center, Guangzhou, Guangdong, China; 6 Affiliated Cancer Hospital and Institute of Guangzhou Medical University, Guangzhou Medical University, Guangzhou, Guangdong, China; Mayo Clinic Cancer Center, UNITED STATES OF AMERICA

## Abstract

Renal cell carcinoma (RCC) is a common urological malignancy, yet the cytokine landscape of the tumor microenvironment, especially in early-stage tumors, remains poorly defined. Using antibody arrays, 174 tumor-related cytokines were measured in 20 sets of normal, para-cancerous, and tumor tissues from early-stage RCC patients. Selected cytokines were validated by ELISA. Functional enrichment was analyzed via DAVID, while transcriptional expression, survival outcomes, and immune infiltration were assessed using GEPIA, Kaplan-Meier Plotter, and TIMER databases. Thirty-four differentially expressed cytokines were identified in tumors versus controls, enriched in cytokine signaling, TNF, PI3K-Akt, Ras, HIF-1, and Toll-like receptor pathways. ELISA confirmed reduced IL-2, IL-15, IGFBP-2, HGF, ErbB3, and FLT3LG, and increased CCL4, CXCL9, and Angiopoietin-2 in tumors. Serum levels of HGF, ErbB3, and CCL4 were decreased in RCC patients, while urinary ErbB3 was also reduced. IL-2, IL-15, and CCL4 were upregulated in T1-T2 metastatic tumors. Several cytokines correlated with poor survival, except ErbB3, which associated with improved outcomes. Several cytokine-related genes, including CXCL9 and CCL4, correlated with inferred immune cell infiltration patterns. These findings suggest that early-stage RCC may be associated with a cytokine expression profile characterized by reduced immune-stimulating factors and elevated pro-angiogenic and inflammatory signals. Urinary ErbB3 emerged as a candidate non-invasive biomarker requiring further validation.

## Introduction

RCC accounts for approximately 3% of all adult malignancies and remains one of the most common urologic cancers worldwide [1]. Global RCC incidence has continued to rise over the past decade, with more than 300,000 new cases and approximately 150,000 RCC-related deaths reported annually [2]. In China, RCC represents a growing public health burden and is associated with nearly 23,400 deaths each year [3]. Although patients with localized RCC often achieve favorable outcomes following surgical resection, more than 30% of patients present with locally advanced or metastatic disease at diagnosis, which is associated with substantially reduced survival despite advances in targeted therapies and immunotherapy [4]. In addition to its clinical heterogeneity, RCC is characterized by complex interactions between tumor cells, stromal components, and immune signaling pathways within the tumor microenvironment, highlighting the need to better understand the biological mechanisms that contribute to tumor progression and disease aggressiveness.

RCC is considered one of the most immune-infiltrated solid tumor types, and increasing evidence suggests that the renal tumor microenvironment plays a central role in disease progression and therapeutic response [5]. However, immune cell infiltration in RCC does not necessarily reflect effective anti-tumor immunity. Integrative molecular and immune profiling studies have demonstrated that highly immune-infiltrated RCC tumors can exhibit aggressive clinical behavior and are frequently associated with immunosuppressive microenvironmental features [6,7]. Although lymphocytes are often abundant within RCC tissues, their cytotoxic activity may be impaired by complex interactions among tumor cells, stromal components, cytokines, and other immunoregulatory factors within the tumor microenvironment.

Cytokines and chemokines produced by tumor cells, stromal cells, and infiltrating immune populations contribute to the complex immunoregulatory landscape of RCC by influencing immune cell recruitment, angiogenesis, inflammatory signaling, and therapeutic response. Several cytokines have been implicated in RCC progression and immune modulation, including IL-10, which has been associated with metastatic disease and immunosuppressive macrophage activity [8,9]. In addition, hypoxia-related signaling pathways involving HIF and TGF-β have been linked to tumor progression and metastatic behavior in RCC [10,11], while IL-6-mediated signaling has been associated with resistance to tyrosine kinase inhibitors through activation of AKT-mTOR, NF-κB, and VEGF-related pathways [12]. Cytokine-directed immunotherapies such as high-dose IL-2 have also demonstrated that modulation of immune signaling pathways can influence clinical outcomes in metastatic RCC, despite limitations related to treatment-associated toxicity [13]. Collectively, these findings highlight the importance of cytokine-mediated communication within the RCC tumor microenvironment. However, the cytokine alterations associated with early-stage RCC and their relationship to tumor progression remain incompletely characterized.

To further characterize cytokine-associated signaling patterns within the RCC tumor microenvironment, we performed exploratory cytokine profiling of early-stage RCC tissues using multiplex antibody arrays targeting 174 cytokine-related proteins. Differentially expressed cytokines identified in tumor tissues were subsequently evaluated in independent tissue, serum, and urine cohorts using ELISA-based analyses, and complementary transcriptomic and immune infiltration analyses were performed using TCGA-KIRC and TIMER datasets. Across these analyses, RCC tissues demonstrated altered expression patterns involving cytokines associated with angiogenesis, inflammatory signaling, immune regulation, and leukocyte recruitment. In addition, altered expression of molecules previously implicated in RCC progression and metastatic behavior, including HGF, ErbB3, and IGFBP-2, was observed [14–16]. However, variability and incomplete concordance between tissue protein, circulating protein, and transcriptomic datasets were also observed, consistent with the complex and dynamic nature of cytokine biology within the tumor microenvironment. Collectively, this study provides an exploratory characterization of cytokine-related alterations in early-stage RCC and highlights the need for larger, mechanistic, and longitudinal studies to further define the biological and clinical significance of these immune signaling patterns.

## Materials and Methods

### Patient cohorts and clinical sample collection

The use of human tumor tissue, serum, and urine samples in this study was conducted in accordance with the Declaration of Helsinki, relevant institutional guidelines, and applicable regulations, and was approved by the Clinical Research and Experimental Animal Ethics Committee of The First Affiliated Hospital of Sun Yat-Sen University (Guangzhou, China). Ethical approval was granted under reference number [2021]144 on 15/03/2021 and was subsequently extended under reference number [2023]260 on 14/07/2023. Written informed consent was obtained from all participants prior to inclusion in the study, and patient privacy rights were strictly protected.

Patients with pathologically confirmed RCC were enrolled through the Department of Urology at The First Affiliated Hospital of Sun Yat-Sen University. Clinical cohorts included patients with predominantly localized early-stage RCC (T1–T2). Available clinicopathological characteristics included patient age, sex, tumor size, T stage, and metastatic status. The tissue cohorts consisted predominantly of clear cell RCC cases, and most tumors with available pathological grading information were classified as grade I–II. Clinical characteristics are summarized in Table 1.

**Table 1:** Clinical and sample characteristics of the RCC study cohorts.

Characteristic	Cohort A	Cohort B	Cohort C
Tissue samples, n	20	38	19
Serum samples, n	–	–	19
Urine samples, n	–	–	19
Age, y			
<60	14	27	Tissue: 12; Serum: 19; Urine: 19
≥60	6	11	Tissue: 7; Serum: 0; Urine: 0
Sex			
Male	16	27	Tissue: 14; Serum: 6; Urine: 16
Female	4	11	Tissue: 5; Serum: 13; Urine: 3
T stage			
T1	15	31	Tissue: 15
T2	5	7	Tissue: 4
Metastatic status			
No	20	36	Tissue: 17
Yes	0	2	Tissue: 2
Tumor size, median (range), cm*	5.1 (2.0–10.0)	4.5 (2.3–10.0)	Tissue: 4.3 (2.0–10.0)

*Tumor size was summarized using the largest recorded tumor dimension. For Cohort C, characteristics are reported separately for the tissue, serum, and urine sample sets.

**Cohort A** consisted of 20 fresh RCC tumor and corresponding para-cancerous tissue specimens collected following radical nephrectomy between 01/05/2021 and 31/12/2021 under ethical approval [2021]144. These samples were analyzed using antibody cytokine microarrays.

**Cohort B** included 38 primary RCC tumor tissues and para-cancerous tissues collected through surgical resection between 15/08/2023 and 31/05/2024 under ethical approval [2023]260. These samples were used for ELISA-based validation analyses.

**Cohort C** comprised serum and urine samples collected from 19 RCC patients and 19 healthy individuals between 15/08/2023 and 31/05/2024 under ethical approval [2023]260. These samples were also analyzed using ELISA.

Para-cancerous tissue was defined as tissue located at least 0.5 cm from the tumor border, whereas normal kidney tissue was collected at least 1 cm from the tumor border. All RCC diagnoses were confirmed by pathological examination of surgical specimens.

### Cytokine array analysis

Sandwich-based membrane antibody arrays (AAH-CYT-2000; RayBio® Human Cytokine Antibody Arrays C6, C7, and C8; RayBiotech Life, Inc., Peachtree Corners, GA, USA) were used to semi-quantitatively measure the expression levels of 174 human cytokines. Normal, para-cancerous, and tumor tissues from RCC patients were homogenized in RayBio® Lysis Buffer (AA-LYS, RayBiotech, Peachtree Corners, GA, USA) supplemented with a protease inhibitor cocktail (AA-PI, RayBiotech, Peachtree Corners, GA, USA). Tissue homogenates were centrifuged at 1,500 × g to remove cellular debris, and all sample preparation procedures were performed at low temperatures.

Equal amounts of total protein lysate were incubated with blocked antibody array membranes according to the manufacturer’s protocol. Following sample incubation, membranes were sequentially incubated with biotinylated antibody cocktails and HRP-conjugated streptavidin, and chemiluminescent signals were detected using enhanced chemiluminescence reagents. Membranes were exposed using a chemiluminescence imaging system, and signal intensities were quantified by densitometric analysis.

For each cytokine, duplicate spot intensities were averaged following background subtraction. Duplicate spots and internal positive controls were used to assess assay consistency and normalization across membranes. Signal intensities were normalized to positive control spots on each membrane according to the manufacturer’s recommended normalization procedure.

Differential expression analysis was performed using the limma package in R. Normalized cytokine expression data were fitted to a linear model using lmFit, followed by empirical Bayes moderation to improve variance estimation across cytokines. Overall differences among normal, para-cancerous, and tumor tissues were evaluated using moderated F-statistics, and pairwise comparisons were performed using moderated t-statistics generated through customized contrast matrices. False discovery rate (FDR) correction was applied using the Benjamini-Hochberg procedure, and cytokines with FDR < 0.05 were considered significantly differentially expressed. Candidate cytokines for downstream analyses were selected based on statistical significance, fold change, and biological relevance to RCC-associated signaling pathways. For the representative cytokine comparisons shown in Fig 2, normality was assessed using the Shapiro–Wilk test, and group differences were evaluated using the Kruskal–Wallis H test followed by pairwise Mann–Whitney U (Wilcoxon rank-sum) tests with Benjamini–Hochberg correction for multiple comparisons.

Differential cytokine expression patterns were visualized using hierarchical clustering heatmaps generated with the R package pheatmap based on Euclidean distance and average linkage clustering. Functional pathway enrichment analysis of differentially expressed cytokines was performed using the DAVID Bioinformatics Resources database.

### Enzyme-linked immunosorbent assay (ELISA)

The concentrations of selected cytokines in serum, urine, and tissue lysates were measured using sandwich-based human ELISA kits specific for Angiopoietin-2 (ELH-Angiopoietin2), RANTES/CCL5 (ELH-RANTES-1), PDGF-AA (ELH-PDGFAA), IL-2 (ELH-IL2), IL-15 (ELH-IL15), MIP-1β/CCL4 (ELH-MIP1b), MIG/CXCL9 (ELH-MIG), IGFBP-2 (ELH-IGFBP2), HGF (ELH-HGF), ErbB3 (ELH-ErbB3), and Flt-3 Ligand (ELH-FLT3L) (RayBiotech Life, Inc., Peachtree Corners, GA, USA). Assays were performed according to the manufacturer’s protocols.

Briefly, standards and diluted samples were added to 96-well microplates pre-coated with cytokine-specific capture antibodies and incubated at room temperature. After washing, biotinylated detection antibodies and HRP-conjugated streptavidin were sequentially added. Signal development was performed using TMB substrate solution, and absorbance was measured at 450 nm using a microplate reader.

Standard curves were generated using serial dilutions of recombinant protein standards provided in each kit and fitted using a four-parameter logistic (4PL) regression model. Cytokine concentrations were calculated by interpolation from best-fit standard curves after subtraction of blank control values. All samples and standards were analyzed in duplicate. Mean absorbance values were used for downstream statistical analyses. The standard concentration series and corresponding curve-fit statistics (R²) for each ELISA are provided in S1 Table.

### Kaplan–Meier survival and TCGA gene expression analyses

Gene Expression Profiling Interactive Analysis (GEPIA; http://gepia.cancer-pku.cn/) was used to analyze RNA sequencing expression data from tumor and normal tissues within the TCGA Kidney Renal Clear Cell Carcinoma (TCGA-KIRC) dataset. Differential gene expression analyses were performed using a cutoff threshold of |log2FC| > 2 and p < 0.05.

Kaplan–Meier Plotter (http://kmplot.com/analysis/) was used to evaluate the prognostic significance of selected cytokine-related genes in TCGA-KIRC patients using the Pan-cancer RNA-seq dataset. Genes were queried using official gene symbols. Overall survival analyses were performed by stratifying patients into high- and low-expression groups using the platform’s automatically selected best cutoff. The expression cutoff values used were 3,147 for ANGPT2, 2 for IL2, 250 for IL15, 2,340 for CXCL9, 382 for CCL4, 521 for HGF, 3,076 for ERBB3, 69 for FLT3LG, and 708 for IGFBP2. Hazard ratios (HRs), 95% confidence intervals (CIs), and log-rank p-values were calculated to assess survival differences between groups.

### Immune Infiltration Analysis

The Tumor Immune Estimation Resource (TIMER) V2.0 (https://cistrome.shinyapps.io/timer/) was used to evaluate the association between cytokine-related gene expression and immune cell infiltration within the TCGA-KIRC dataset. Associations between the expression of cytokine-related genes (CD14, AXL, CCL4, CXCL9, and CCL5) and inferred immune cell infiltration levels were analyzed using Spearman correlation analysis with tumor purity adjustment. Statistical significance was defined as P < 0.05. Correlation coefficients and corresponding p-values were obtained directly from the TIMER V2.0 platform. TIMER estimates the abundance of multiple tumor-infiltrating immune cell populations using established immune deconvolution algorithms based on bulk RNA sequencing data.

### Statistical Analysis

Data are presented as mean ± standard deviation (SD). Statistical analyses were performed using SPSS software (SPSS 13.0 for Windows; SPSS Inc., Chicago, IL, USA) and R. Normality was assessed using the Shapiro–Wilk test. For comparisons among multiple groups, one-way analysis of variance (ANOVA) followed by Tukey’s HSD post hoc test was used when all groups were normally distributed. When normality assumptions were not met, the Kruskal–Wallis H test was used, followed by pairwise Mann–Whitney U (Wilcoxon rank-sum) tests with Benjamini–Hochberg correction for multiple comparisons. For two-group comparisons, Student’s t-test was used for normally distributed data, whereas the Mann–Whitney U test was used when normality assumptions were not met. TCGA gene expression data were analyzed using the Wilcoxon rank-sum test. TIMER immune infiltration analyses were performed using Spearman correlation analysis with tumor purity adjustment. Statistical significance was defined as p < 0.05 unless otherwise specified.

## Results

### A distinct cytokine protein signature characterizes early-stage RCC tissues

To characterize cytokine alterations associated with localized RCC, antibody arrays measuring 174 cytokine-related proteins were performed using tumor, para-cancerous, and normal kidney tissue samples obtained from 20 RCC patients. Among the proteins analyzed, 34 cytokines were identified as differentially expressed between tumor tissues and both para-cancerous and normal control tissues.

Hierarchical clustering analysis of the antibody array protein data demonstrated that tumor tissues exhibited a distinct cytokine expression profile compared with para-cancerous and normal kidney tissues (Fig 1A). Overall, the differentially expressed cytokines reflected broader alterations in angiogenesis-associated signaling, inflammatory responses, immune cell recruitment, and immune regulatory pathways within the RCC tumor microenvironment. Cytokines associated with angiogenesis, inflammatory signaling, and immune cell recruitment, including VEGF, PDGF-AA, sgp130, CD14, ICAM-1, ICAM-2, PIGF, AXL, Angiopoietin-2, EGFR, CXCL9, IP-10/CXCL10, CCL4, CCL5, MMP-9, TIMP-1, Siglec-5/CD170, sTNF RII, Fas/TNFRSF6, and LAP, were generally elevated in tumor tissues. In contrast, several cytokines involved in immune activation and growth regulation, including CNTF, IGFBP-2, HGF, EGF, TGF-β1, M-CSF, TNF-β, FLT3LG, ErbB3, IL-2, IL-15, IL-1ra, IL-7, and IL-13, showed reduced expression in tumor tissues relative to control tissues.

**Fig 1:**
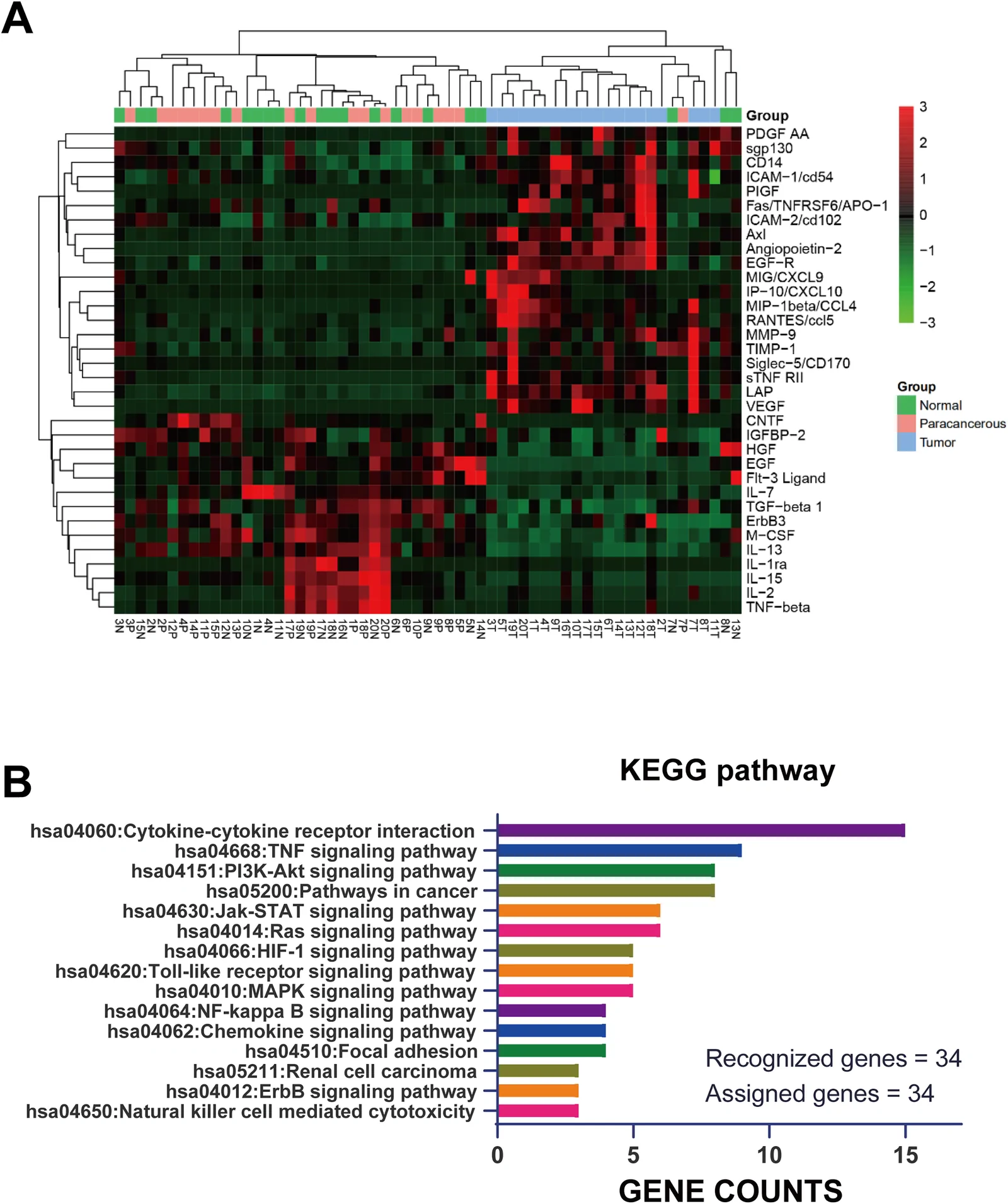
Differential cytokine protein profile and pathway enrichment in early-stage RCC tissues. (A) Hierarchical clustering heatmap of 34 differentially expressed cytokines identified by antibody array analysis of RCC tumor, para-cancerous, and normal kidney tissue samples. Differential expression was determined using limma-based linear modeling with Benjamini–Hochberg false discovery rate (FDR) correction (FDR < 0.05). Each column represents one tissue sample, and each row represents one cytokine. The annotation bar above the heatmap indicates sample group classification (normal, para-cancerous, or tumor). Relative cytokine abundance is represented by color intensity, with red indicating higher expression and green indicating lower expression relative to the normalized signal intensity scale. Clustering analysis was performed using Euclidean distance and average linkage. **(B)** KEGG pathway enrichment analysis of the 34 differentially expressed cytokines identified by antibody array profiling. The cytokine set was selected based on statistical significance, fold change, and biological relevance following differential expression analysis. Bars indicate the number of cytokines assigned to each enriched signaling pathway. Pathway enrichment was performed using DAVID based on the set of differentially expressed cytokines identified in panel A. Pathway enrichment was performed using DAVID based on the set of differentially expressed cytokines identified in panel A. Enriched pathways included cytokine–cytokine receptor interaction, TNF signaling, PI3K-Akt signaling, Ras signaling, HIF-1 signaling, and Toll-like receptor signaling pathways.

KEGG pathway enrichment analysis of the differentially expressed cytokines demonstrated significant enrichment in pathways associated with cytokine signaling, inflammation, hypoxia, and tumor progression, including cytokine–cytokine receptor interaction, TNF signaling, PI3K-Akt signaling, Ras signaling, HIF-1 signaling, and Toll-like receptor signaling pathways (Fig 1B). These pathway analyses further supported the presence of broad immune and inflammatory signaling alterations in early-stage RCC tissues.

Representative cytokines identified from the antibody array dataset are shown in Fig 2. Quantified array signal intensities and representative membrane images demonstrated increased expression of Angiopoietin-2, CCL4, RANTES/CCL5, and CXCL9 in tumor tissues compared with normal tissues, whereas IL-2, IL-15, ErbB3, HGF, and FLT3LG showed reduced expression in tumor tissues. IL-2 showed a similar trend toward reduced expression in tumor tissues, although the difference did not reach statistical significance. These findings further illustrate the distinct cytokine protein landscape observed in early-stage RCC tissues. These representative cytokines were selected to illustrate the heterogeneous cytokine expression patterns identified across the antibody array dataset.

**Fig 2:**
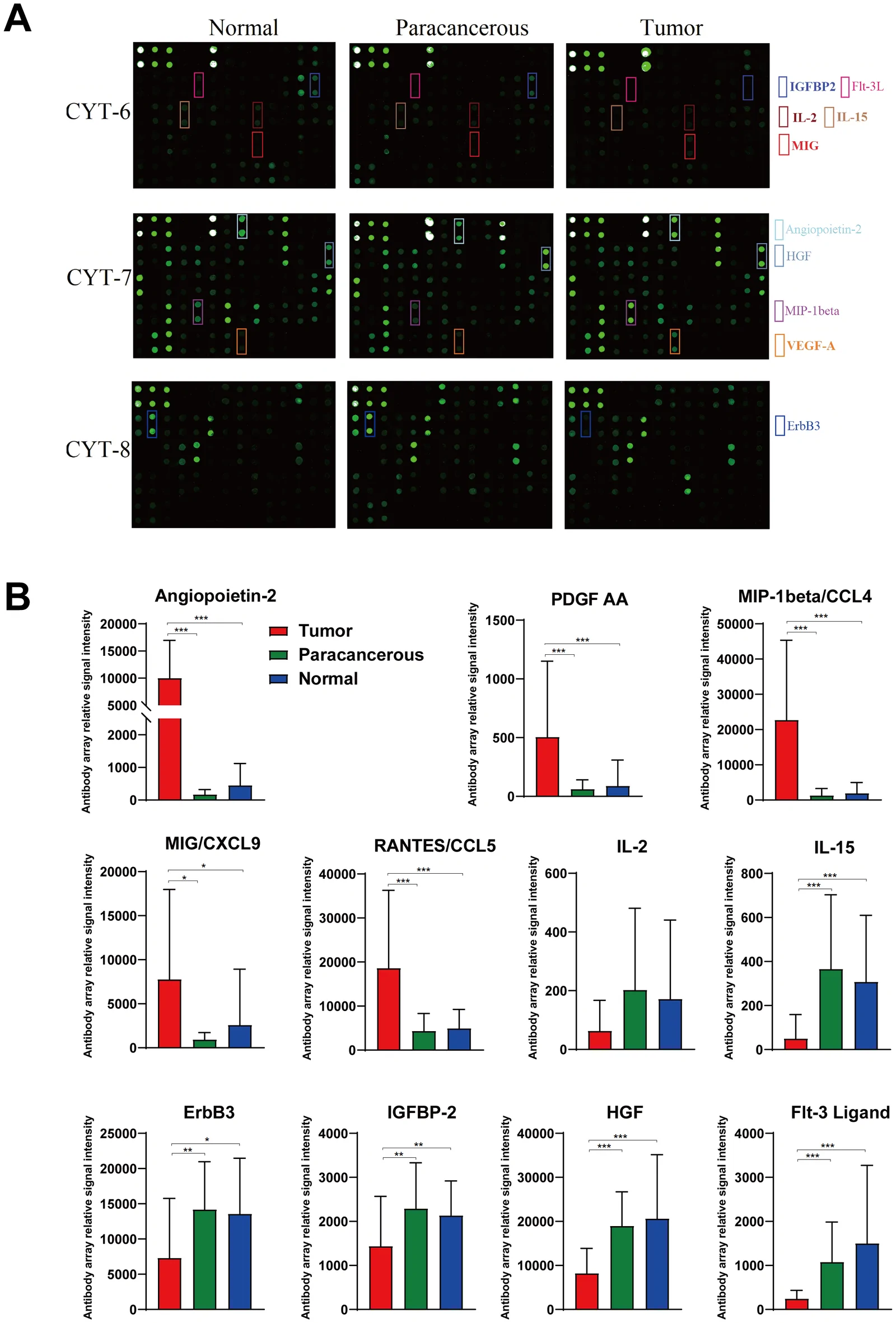
Representative cytokine alterations identified by antibody array analysis in RCC tissues. (A) Representative antibody array membrane images from normal (left), para-cancerous (middle), and tumor (right) tissue samples analyzed using RayBio® Human Cytokine Antibody Arrays C6, C7, and C8. Colored boxes indicate the locations of representative cytokines shown in panel B. (B) Quantified signal intensities of representative cytokines identified from the antibody array dataset. Bar graphs show normalized cytokine expression levels in tumor, para-cancerous, and normal kidney tissue samples. Cytokines associated with angiogenesis and inflammatory signaling, including Angiopoietin-2, CCL4, RANTES/CCL5, and CXCL9, showed increased expression in tumor tissues, whereas IL-15, ErbB3, HGF, and FLT3LG showed decreased expression relative to control tissues. IL-2 showed a trend toward decreased expression in tumor tissues, although the difference did not reach statistical significance. Statistical significance annotations indicate comparisons among tissue groups as described in the Methods section. Statistical significance is indicated as follows: *P < 0.05, **P < 0.01, and ***P < 0.001.

### Validation of differentially expressed cytokines using ELISA

To validate the cytokine alterations identified by antibody array profiling, selected cytokines were further analyzed using ELISA in an independent RCC tissue cohort (Cohort B). Eleven candidate cytokines were selected for validation based on fold-change magnitude and biological relevance to angiogenesis, inflammatory signaling, and immune regulation.

ELISA analysis confirmed differential expression patterns for 10 of the 11 evaluated cytokines (Fig 3). Angiopoietin-2, CCL4, and CXCL9 showed significantly increased expression in RCC tumor tissues relative to normal tissues, whereas IL-2, IL-15, IGFBP-2, HGF, ErbB3, FLT3LG, and PDGF-AA showed reduced expression in tumor tissues. In contrast, RANTES/CCL5 did not demonstrate a statistically significant difference between tissue groups in the validation cohort. Overall, these findings demonstrated partial concordance between the multiplex antibody array and ELISA analyses while also highlighting variability among individual cytokine measurements across independent sample cohorts.

**Fig 3:**
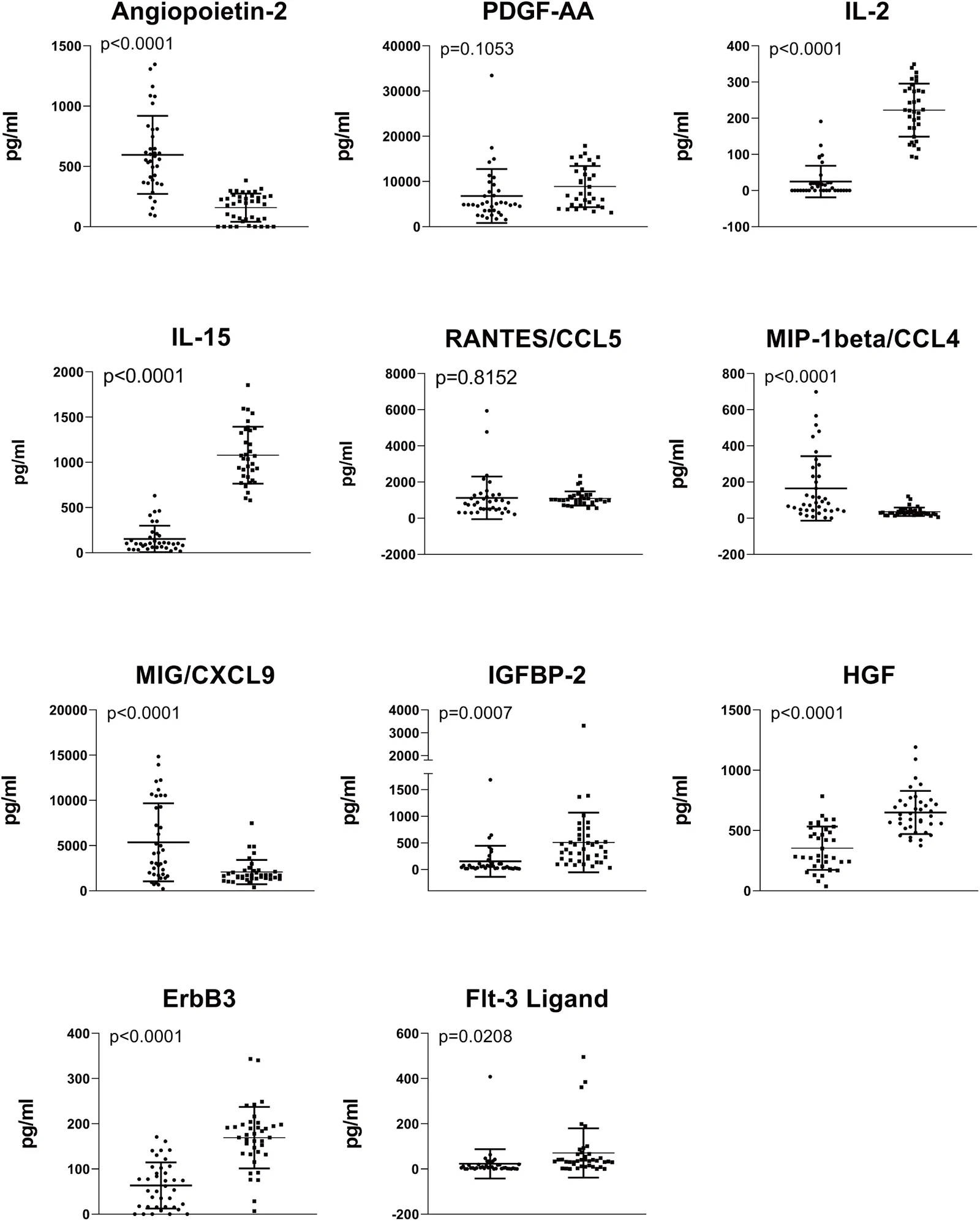
ELISA validation of differentially expressed cytokines in RCC tissues.

Expression levels of selected cytokines were measured by ELISA in RCC tumor and normal kidney tissue samples from the independent validation cohort (Cohort B). Each panel represents one cytokine identified from the antibody array discovery analysis. Round dots (left side of each panel) represent tumor tissue samples, and square dots (right side of each panel) represent kidney tissue samples. Individual symbols represent single patient samples. Angiopoietin-2, CCL4, and CXCL9 showed increased expression in tumor tissues, whereas IL-2, IL-15, IGFBP-2, HGF, ErbB3, FLT3LG, and PDGF-AA showed reduced expression relative to normal tissues. RANTES/CCL5 did not show a statistically significant difference between groups. P values are shown within individual panels. Statistical significance is indicated as follows: *P < 0.05, **P < 0.01, and ***P < 0.001.

### TCGA transcriptomic analyses demonstrate stage- and metastasis-associated cytokine expression patterns

To further examine the clinical relevance of cytokines identified in the protein profiling analyses, transcriptomic data from the TCGA-KIRC dataset were analyzed using GEPIA and Kaplan–Meier Plotter databases. mRNA expression patterns of selected cytokines were evaluated according to tumor stage and metastatic status.

Among the evaluated cytokines, IL-2, IL-15, and CCL4 demonstrated significantly increased mRNA expression in metastatic T1–T2 RCC tumors compared with non-metastatic tumors of the same stage (Fig 4). This trend was not observed in T3–T4 tumors. CXCL9 showed similar expression trends but with greater variability between patient groups.

**Fig 4:**
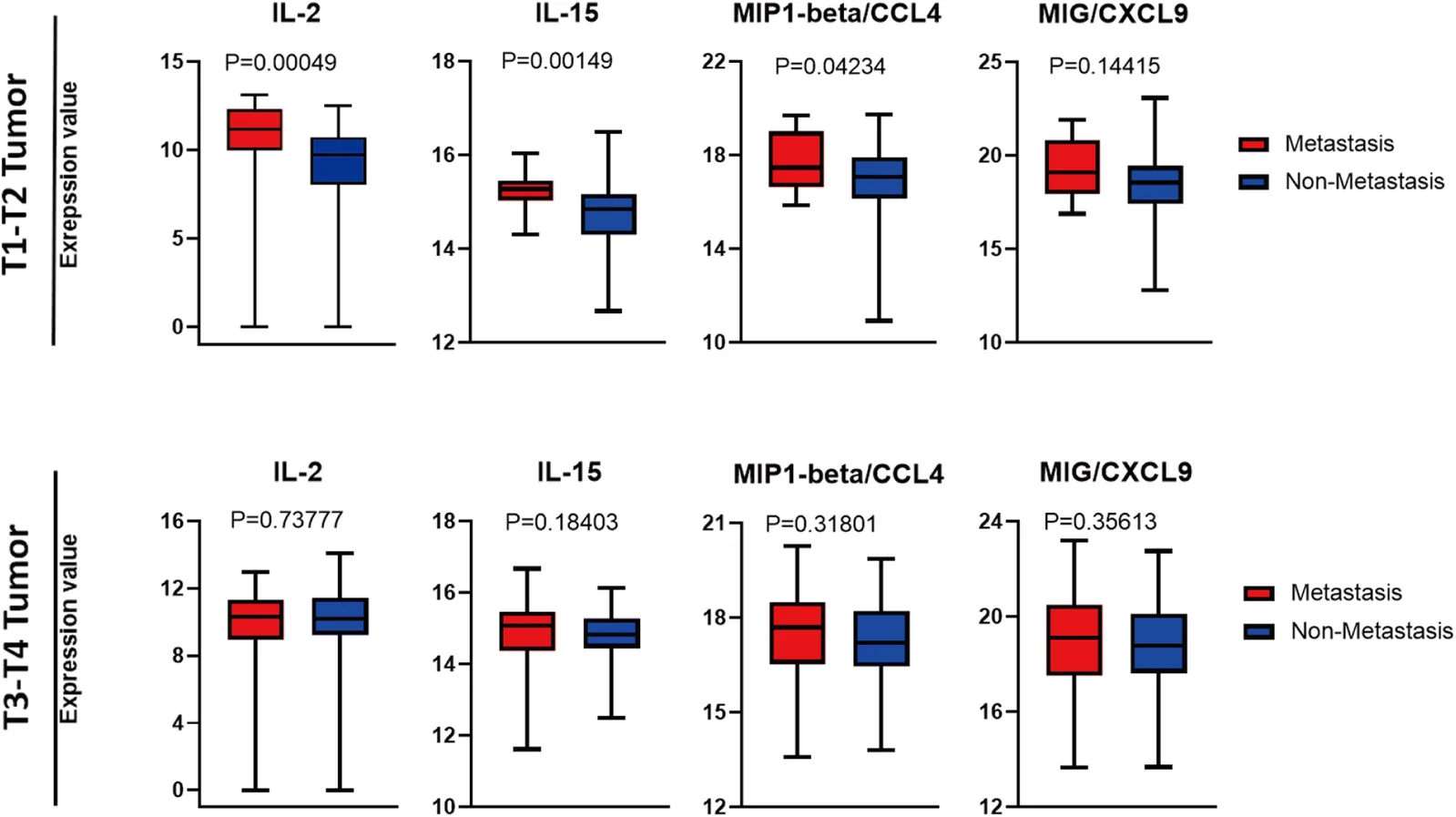
TCGA-KIRC transcriptomic analysis of selected cytokine-related genes in RCC.mRNA expression levels (log2(FPKM+1)) of IL-2, IL-15, CCL4, and CXCL9 in metastatic and non-metastatic clear cell RCC (ccRCC) tumors from the TCGA-KIRC dataset. Analyses were stratified by tumor stage (T1–T2 and T3–T4). Each dot represents an individual patient sample. Data represent transcript-level expression derived from TCGA-KIRC RNA sequencing datasets. Comparisons were performed using TCGA transcriptomic data obtained through GEPIA and related bioinformatic platforms. Statistical significance is indicated within individual panels.

Additional TCGA analyses demonstrated elevated transcript expression of Angiopoietin-2, CXCL9, and CCL4 in RCC tissues, whereas IGFBP-2 expression was reduced relative to normal tissues (S1 Fig).

Kaplan–Meier survival analyses identified several cytokine-related genes associated with patient prognosis in RCC (S2 Fig). Higher expression of IL-2, IL-15, CCL4, HGF, FLT3LG, and IGFBP2 was associated with poorer overall survival, whereas elevated ErbB3 expression was associated with improved survival outcomes.

Importantly, these transcriptomic findings were not always consistent with the protein-level observations identified by antibody array and ELISA analyses, highlighting the complex relationship between cytokine mRNA expression and functional protein abundance within the tumor microenvironment. Because cytokine signaling is influenced by dynamic secretion patterns, post-transcriptional regulation, and differences between tissue and circulating compartments, these transcriptomic analyses were interpreted primarily as complementary contextual analyses rather than direct validation of the protein-level findings.

### Cytokine-related transcripts are associated with immune cell infiltration patterns in RCC

Because several cytokines identified in the antibody array analyses are known regulators of leukocyte recruitment and immune signaling within the tumor microenvironment [17], we next examined whether their transcript expression was associated with immune cell infiltration patterns in RCC using the TIMER database.

Transcript expression of several cytokine-related genes demonstrated positive correlations with inferred immune cell infiltration within the TCGA-KIRC dataset (Fig 5; S3 Fig). Fig 5 highlights the strongest cytokine–immune cell associations identified in the analysis, whereas the complete correlation dataset is presented in S3 Fig. Among the strongest associations observed, CXCL9, CCL4, and CCL5 expression positively correlated with CD8 + T-cell infiltration, with correlation coefficients of 0.68, 0.59, and 0.69, respectively. Dendritic cell infiltration also showed positive associations with CXCL9, CCL4, CCL5, and CD14 expression. In addition, macrophage infiltration correlated with CD14 and AXL expression, whereas neutrophil infiltration was associated with CD14, AXL, CXCL9, and CCL4 expression. CXCL9 expression also demonstrated a moderate positive correlation with B-cell infiltration.

**Fig 5:**
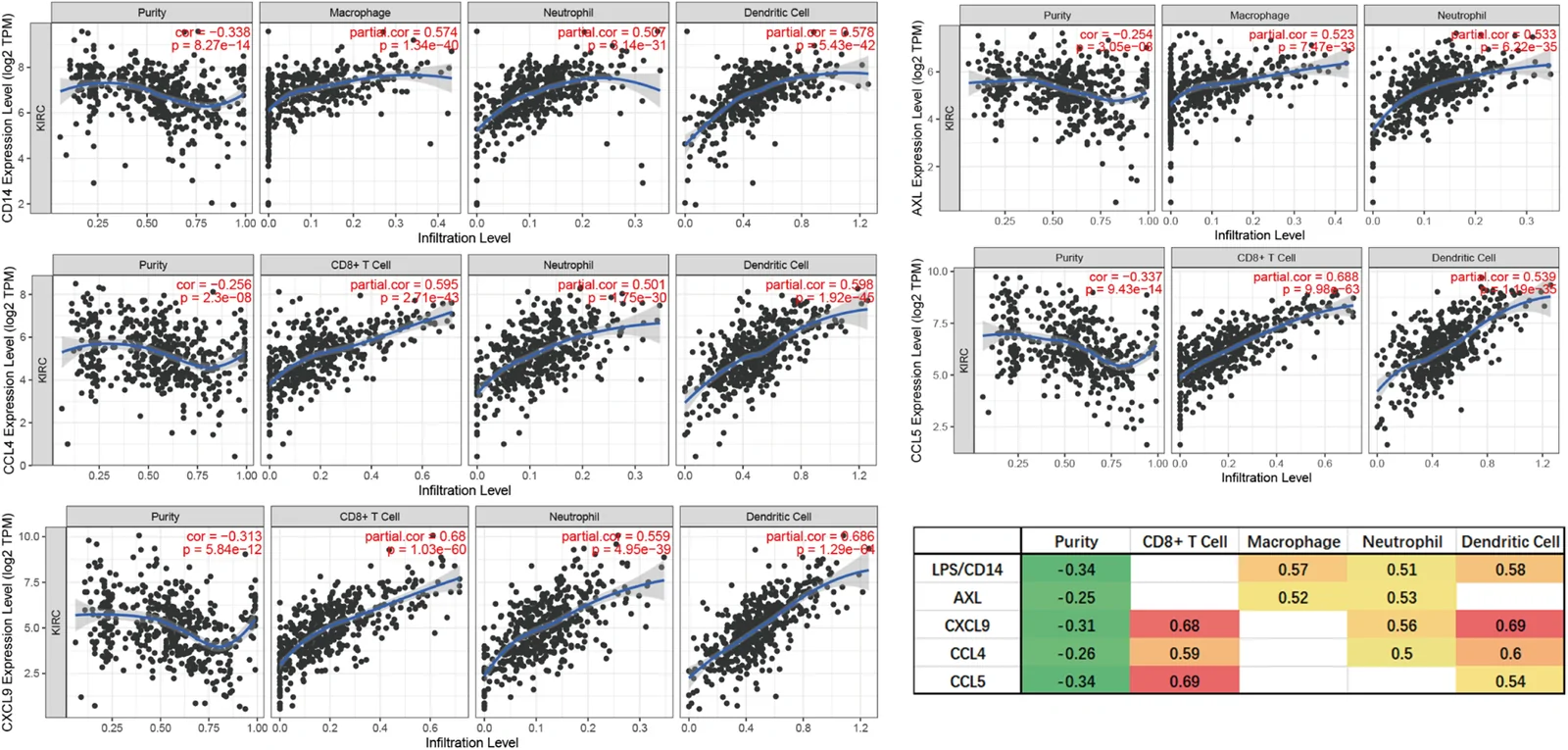
Correlation between cytokine-related gene expression and immune cell infiltration in RCC.

These findings suggest that cytokine-related transcriptional programs identified in RCC may be associated with broader immune microenvironment remodeling. Because these analyses were derived from transcriptomic datasets and computational immune deconvolution approaches, the observed associations should be interpreted within the context of RNA-level immune infiltration estimates.

Heatmap showing the strongest correlations between cytokine-related gene expression and inferred immune cell infiltration within the TCGA-KIRC dataset using TIMER V2.0. Rows represent immune cell populations and columns represent cytokine-related genes identified from the antibody array analyses. Correlation coefficients derived from Spearman correlation analysis with tumor purity adjustment are displayed within individual heatmap cells and represented by the continuous color gradient. Positive correlations indicate associations between increased transcript expression and higher estimated immune cell infiltration levels. Additional cytokine–immune cell correlation analyses are provided in S3 Fig.

### Altered serum and urine cytokine levels in patients with localized RCC

To further explore whether selected cytokine alterations identified in RCC tissues could also be detected in circulating or urinary compartments, serum and urine samples from Cohort C were analyzed by ELISA. In serum, HGF, ErbB3, and MIP-1β/CCL4 levels were significantly decreased in patients with localized ccRCC compared with healthy controls, whereas CXCL9 levels were not significantly different. In urine samples, ErbB3 and MIP-1β/CCL4 levels were significantly reduced in ccRCC patients, while HGF and CXCL9 showed no significant differences (Fig 6). These findings demonstrate that selected cytokine-related alterations identified in RCC tissues may also be detectable in serum or urine; however, the degree and direction of these changes varied across biological compartments.

**Fig 6:**
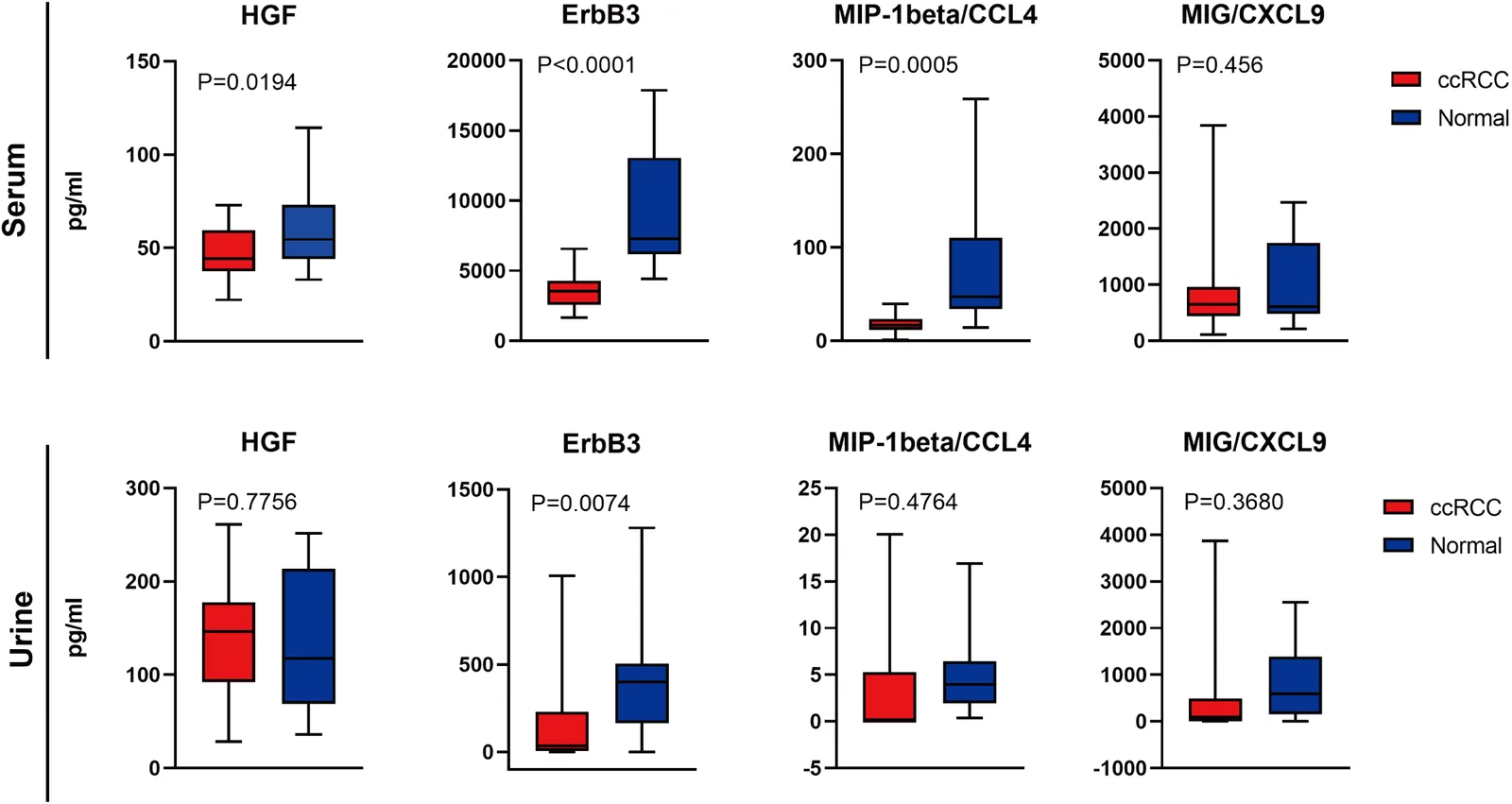
ELISA validation of selected cytokines in serum and urine samples from patients with clear cell RCC. Serum (top row) and urine (bottom row) levels of HGF, ErbB3, MIP-1β/CCL4, and MIG/CXCL9 were measured by ELISA in patients with ccRCC and healthy controls. Box plots represent the median, interquartile range, and minimum-to-maximum values. Statistical significance is indicated as follows: *P < 0.05, **P < 0.01, and ***P < 0.001. P values indicate statistical significance between groups.Top of Form.

## Discussion

RCC is characterized by a highly immune-infiltrated yet functionally complex tumor microenvironment [18] in which cytokine-mediated signaling plays important roles in angiogenesis, inflammatory regulation, immune cell recruitment, and immune suppression [6,19]. Previous studies have reported altered cytokine patterns in RCC tissues, including reduced expression of T cell-associated cytokines such as IL-2 and IFN-γ alongside elevated inflammatory cytokines including IL-6 and IL-10 [20]. In addition, hypoxia-associated signaling within RCC has been linked to inflammatory cytokine production and activation of pathways involved in tumor progression and metastatic behavior, including STAT3 and PI3K-Akt signaling [11]. Although RCC tumors frequently demonstrate substantial immune cell infiltration, increasing evidence suggests that these infiltrating immune populations often exist within an immunoregulatory microenvironment that may limit effective anti-tumor immune responses [6]. Consistent with these observations, our exploratory cytokine profiling analyses identified broad alterations in cytokine-associated signaling pathways related to angiogenesis, inflammatory signaling, and immune regulation in early-stage RCC tissues.

Our exploratory cytokine profiling analyses identified altered expression patterns involving angiogenesis-associated, inflammatory, and immune regulatory signaling molecules in early-stage RCC tissues. Cytokines and related signaling proteins associated with angiogenesis and vascular remodeling, including Angiopoietin-2, VEGF, PIGF, MMP-9, and TIMP-1, demonstrated increased expression in RCC tumor tissues, whereas several cytokines involved in immune activation and lymphocyte regulation, including IL-2, IL-15, IL-13, IL-7, and M-CSF, showed reduced expression. In addition, elevated expression of inflammatory chemokines and immune-associated molecules such as CD14, AXL, CXCL9, CCL4, and CCL5 was observed, suggesting broader alterations in immune cell recruitment and inflammatory signaling within the RCC tumor microenvironment. Several molecules previously implicated in tumor progression and metastatic signaling, including HGF, IGFBP-2, ErbB3, and FLT3LG, unexpectedly demonstrated lower expression in early-stage RCC tissues relative to adjacent control tissues. Notably, selected findings from the discovery cohort, including alterations in Angiopoietin-2, CCL4, CXCL9, HGF, and ErbB3, were further evaluated in independent tissue, serum, or urine validation analyses. Because the present study primarily evaluated localized T1–T2 RCC tumors, these findings may reflect stage-specific differences in cytokine-associated signaling patterns within the evolving renal tumor microenvironment. Collectively, these observations support the concept that early-stage RCC is associated with complex and heterogeneous alterations in cytokine-related signaling networks rather than uniform activation of a single immune or tumor-promoting pathway [6,18].

Angiogenesis-related signaling molecules represented one of the most prominent cytokine-associated patterns identified in early-stage RCC tissues. Among these, Angiopoietin-2 (Ang2) and VEGF demonstrated increased expression in RCC tumor samples, with ELISA validation further confirming significantly elevated Ang2 levels in tumor tissues relative to normal tissues. Previous studies have identified Ang2 as an important regulator of tumor-associated angiogenesis and vascular remodeling through its interactions with VEGF and endothelial Tie-2 signaling pathway [21,22]. In addition to its vascular effects, Ang2 has also been implicated in inflammatory and immune-associated microenvironment remodeling, including modulation of endothelial activation and leukocyte trafficking [23]. Interestingly, a prior RCC study reported higher Ang2 expression in stage I tumors compared with more advanced stage IV disease [24], which is broadly consistent with the elevated Ang2 expression observed in our early-stage RCC cohort. Collectively, these findings suggest that angiogenesis-associated cytokine signaling alterations may occur early during RCC development and could contribute to vascular and inflammatory remodeling within the tumor microenvironment. However, because the present study was limited to exploratory cytokine profiling analyses, further mechanistic studies are required to clarify the specific functional roles of Ang2 and related angiogenic signaling pathways across different stages of RCC progression.

Chemokine-associated inflammatory signaling represented another prominent feature of the cytokine alterations observed in early-stage RCC tissues. CC and CXC chemokines are important regulators of leukocyte trafficking and immune cell localization within the tumor microenvironment [17]. Previous studies have suggested that chemokine receptor networks involving CXCR3, CXCR6, CCR5, and CCR6 may contribute to immune cell recruitment into RCC tissues [25]. In the present study, elevated expression of CXCL9, CCL4, and CCL5 was identified in RCC tumor tissues by antibody array profiling, while ELISA validation further confirmed significantly increased levels of CCL4 and CXCL9 in tumor tissues relative to normal controls. TCGA transcriptomic analyses also demonstrated elevated expression of CCL4 and CXCL9 in RCC tissues, and higher CCL4 expression was observed in metastatic T1–T2 tumors compared with non-metastatic tumors of the same stage. Previous studies have reported that CXCL9 may participate in immune cell recruitment and inflammatory remodeling within the RCC tumor microenvironment, including interactions involving tumor-associated macrophages and CXCR3-expressing T-cell populations [26]. In addition, CCL4 has been implicated in recruitment of immunoregulatory cell populations, including Foxp3 + regulatory T cells, in several tumor types [25,27]. Consistent with these observations, transcriptomic immune infiltration analyses in the present study demonstrated positive associations between CXCL9 and CCL4 expression and inferred infiltration of CD8 + T cells, dendritic cells, neutrophils, and other immune cell populations within the TCGA-KIRC dataset. However, because these analyses were based on transcriptomic datasets and computational immune deconvolution approaches rather than direct immune phenotyping, the biological significance of these associations should be interpreted cautiously. Collectively, these findings suggest that altered chemokine-associated signaling patterns may contribute to broader immune microenvironment remodeling in RCC.

IL-2 and IL-15 are important regulators of both innate and adaptive immune responses through their roles in activation and maintenance of CD8 + T cells and NK cells [28]. In the present study, reduced IL-2 and IL-15 protein expression was observed in early-stage RCC tumor tissues, which is broadly consistent with previous reports describing decreased levels of these cytokines within localized renal tumors [29]. However, transcriptomic analyses using TCGA-KIRC data demonstrated higher IL-2 and IL-15 mRNA expression in RCC tissues, particularly in metastatic T1–T2 tumors relative to non-metastatic tumors of the same stage, while this trend was less apparent in T3–T4 tumors. In addition, higher IL-2 and IL-15 transcript expression was associated with poorer overall survival in RCC patients. These findings highlight the complex relationship between cytokine protein abundance and transcriptomic expression within the RCC tumor microenvironment. Cytokine expression patterns may vary substantially according to tumor stage, immune cell composition, local inflammatory conditions, secretion dynamics, and post-transcriptional regulatory mechanisms, potentially contributing to discordant findings across tissue protein- and RNA-based analyses [30,31]. Given the dynamic and context-dependent nature of cytokine signaling within the tumor microenvironment, the biological significance of IL-2 and IL-15 alterations in RCC likely depends on multiple interacting immune and microenvironmental factors. Collectively, these findings underscore the importance of cautious interpretation of cytokine-associated transcriptomic and protein-level alterations within the heterogeneous RCC tumor microenvironment.

Several molecules previously implicated in tumor proliferation, metastatic progression, and therapy resistance, including HGF, IGFBP-2, ErbB3, and FLT3LG, demonstrated lower expression in early-stage RCC tissues relative to adjacent control tissues in the present study. HGF-mediated MET signaling has been associated with tumor survival, angiogenesis, and metastatic behavior in multiple malignancies, including RCC [32]. Similarly, IGFBP-2, FLT3 signaling, and ErbB3-associated PI3K/AKT pathway activation have been linked to tumor progression and therapeutic resistance in several cancer types [33–35]. The reduced expression of these molecules in predominantly T1-stage RCC tissues was therefore somewhat unexpected and may reflect differences between early-stage and advanced RCC microenvironmental signaling states. Because many prior studies evaluating these pathways have focused on metastatic or treatment-resistant disease settings, cytokine and growth factor expression patterns observed in localized RCC may not fully mirror those reported in more advanced tumors. In addition, variability between tissue protein abundance, circulating cytokine levels, and transcriptomic expression further highlights the context-dependent and heterogeneous nature of RCC-associated signaling networks. Exploratory serum and urine analyses demonstrated reduced HGF and ErbB3 levels in serum, with ErbB3 also decreased in urine samples from RCC patients. Although these findings suggest that altered ErbB3-associated signaling may be detectable across multiple biological compartments, larger validation studies will be required to determine the potential biological or clinical relevance of these observations.

Several limitations of the present study should be acknowledged. First, the study was designed as an exploratory cytokine profiling analysis of early-stage RCC and was conducted using relatively small patient cohorts, particularly within the validation and body fluid analyses. Given the substantial molecular and immune heterogeneity that characterizes RCC, larger independent cohorts will be necessary to further evaluate the reproducibility and generalizability of the cytokine-associated signaling patterns identified in this study [5,6]. Accordingly, the cytokine patterns identified in this study may not be uniformly represented across all RCC tumors and could reflect the presence of biologically distinct disease subsets that were not specifically evaluated in the current analysis. In addition, although complementary protein-, transcript-, and computational immune infiltration–based analyses were performed, incomplete concordance was observed across tissue protein expression, circulating cytokine measurements, and transcriptomic datasets. Such variability is consistent with the multi-layered regulation of cytokine expression and signaling and may reflect differences in tumor stage, immune cell composition, local inflammatory conditions, secretion dynamics, post-transcriptional regulation, and compartment-specific signaling within the tumor microenvironment [30,31]. Furthermore, immune infiltration analyses were based on transcriptomic deconvolution methods derived from TCGA datasets rather than direct cellular phenotyping, and therefore should be interpreted as indirect estimates of immune microenvironment composition. In addition, the present analyses were based on samples collected at a single clinical time point, limiting assessment of temporal changes in cytokine expression and tumor microenvironment remodeling during RCC progression. Finally, while the present study identified several cytokine-associated alterations linked to angiogenic, inflammatory, and immune regulatory pathways in early-stage RCC, additional mechanistic and longitudinal studies will be required to clarify the functional significance and temporal evolution of these signaling networks during RCC progression. Despite these limitations, the multi-platform approach used in this study provides an exploratory overview of cytokine-associated alterations in localized RCC and may help inform future investigations of RCC tumor microenvironment biology.

In conclusion, the present study identified altered cytokine-associated signaling patterns in early-stage RCC involving angiogenesis-related, inflammatory, and immune regulatory pathways. Although these findings require further validation and mechanistic investigation, this exploratory multi-platform analysis provides additional insight into cytokine-associated tumor microenvironment remodeling in localized RCC.

### Declaration of generative AI and AI-assisted technologies in the writing process

During the preparation of this work, the authors used GPT-4o to assist with language and grammar correction. After using this service, the authors reviewed and edited the content as needed and take full responsibility for the content of the publication.

## Supporting information

S1 Fig Differential mRNA expression of cytokines in renal tumors and normal tissues from the TCGA-KIRC dataset. mRNA expression levels (log2(TPM + 1)) of Angiopoietin-2, IL-2, IL-15, MIG/CXCL9, MIP-1β/CCL4, IGFBP-2, HGF, ErbB3, and Flt-3 Ligand in renal tumors and normal kidney tissues from the TCGA-KIRC dataset. Differential expression analysis was performed using GEPIA with cutoffs of p < 0.05 and |log2FC| > 2.
(TIF)

S2 Fig Kaplan-Meier survival analysis of nine cytokine-related genes in renal cell carcinoma (TCGA-KIRC dataset). Kaplan-Meier plots of nine cytokine-related genes in the TCGA-KIRC dataset generated using the Kaplan-Meier Plotter Pan-cancer RNA-seq dataset. Patients were stratified into high- and low-expression groups using the platform’s automatically selected cutoff settings. Statistical significance was defined as p < 0.05.
(TIF)

S3 Fig Additional immune infiltration correlation analyses of cytokine-related genes in the TCGA-KIRC dataset. Correlations between CD14, AXL, CCL4, CXCL9, and CCL5 expression and immune cell infiltration estimates were evaluated using TIMER V2.0 based on TCGA-KIRC transcriptomic data. Spearman correlation analysis with tumor purity adjustment was used. Correlation coefficients and corresponding p-values are shown.
(TIF)

S1 Table ELISA standard curve concentrations and curve-fit statistics. Standard concentration series, four-parameter logistic (4PL) curve-fitting model, and corresponding R^2^ values for ELISA assays used in the study.
(XLSX)

S1 Data De-identified underlying data for the cytokine antibody array and ELISA analyses. Workbook containing the processed normalized cytokine antibody array data and ELISA concentration data underlying the study analyses, with accompanying data descriptions.
(XLSX)
