# Supplementary material for: Tumor specific cytokine pattern explored in renal cell carcinoma
Source: PLoS One. 2026 Sep 29;21(9):e0358533. doi: 10.1371/journal.pone.0358533 (PMC13623153; doi:10.1371/journal.pone.0358533)
Supplement: Supplementary file 1 [file pone.0358533.s006.docx]

# PLOS ONE Revision Response Document

**Manuscript ID:** PONE-D-26-01255
**Title:** *Tumor Specific Cytokine Pattern Explored in Renal Cell Carcinoma*

# SECTION 1 — JOURNAL REQUIREMENTS

**Journal Requirement 1**

“1. Please ensure that your manuscript meets PLOS ONE's style requirements, including those for file naming. The PLOS ONE style templates can be found at

<https://journals.plos.org/plosone/s/file?id=wjVg/PLOSOne_formatting_sample_main_body.pdf> and

https://journals.plos.org/plosone/s/file?id=ba62/PLOSOne_formatting_sample_title_authors_affiliations.pdf”

**Response**

We thank the editor for this comment. The manuscript has been reformatted according to the PLOS ONE style requirements and templates, including updates to the title page, section headings, figure citations and captions, references, and overall manuscript formatting. File naming conventions were also updated accordingly.

**Journal Requirement 2**

“2. Please include your tables as part of your main manuscript and remove the individual files. Please note that supplementary tables (should remain/ be uploaded) as separate "supporting information" files.”

**Response**

We thank the editor for this comment. All tables were incorporated into the main manuscript file and individual table files were removed. For improved clarity and organization, the original Table 1 was separated into Table 1 (“Clinical characteristics of RCC tissue cohorts (Cohorts A and B).”) and Table 2 (“Clinical characteristics of serum and urine validation cohorts (Cohort C)”). The additional summary table, Table 3 (“Summary of clinical characteristics across RCC patient cohorts”), was also incorporated into the manuscript.

**Journal Requirement 3**

“3. Thank you for stating the following financial disclosure:

‘National Natural Science Foundation of China (No. 81572522 and No. 81202011), Guangdong Innovation Platform Project (2012B090600021), Guangzhou 2024 Annual Special Project on Agricultural and Social Development Science and Technology (2024B03J1332, 2024B03J1249). National Key R&D Program of China (2024YFA1307601, 2024YFA1307602, 2024YFA1307603).’

Please state what role the funders took in the study. If the funders had no role, please state: ‘The funders had no role in study design, data collection and analysis, decision to publish, or preparation of the manuscript.’

If this statement is not correct you must amend it as needed.

Please include this amended Role of Funder statement in your cover letter; we will change the online submission form on your behalf.”

**Response**

We thank the editor for this comment. The Role of Funder statement has now been clarified and included in the revised cover letter. The funders had no role in study design, data collection and analysis, decision to publish, or preparation of the manuscript.

**Journal Requirement 4**

“4. Please note that funding information should not appear in any section or other areas of your manuscript. We will only publish funding information present in the Funding Statement section of the online submission form. Please remove any funding-related text from the manuscript.”

**Response**

We thank the editor for this comment. All funding-related information was removed from the manuscript and will be provided only through the Funding Statement section.

**Journal Requirement 5**

“5. Thank you for stating the following in the Competing Interests section:

‘Ying Luo, Panning Wang, Shuhong Luo and Ruo-Pan Huang are employees of and have a financial stake in RayBiotech, Inc. The authors declare no additional competing interests.’

We note that one or more of the authors are employed by a commercial company: RayBiotech, Inc

1. Please provide an amended Funding Statement declaring this commercial affiliation, as well as a statement regarding the Role of Funders in your study. If the funding organization did not play a role in the study design, data collection and analysis, decision to publish, or preparation of the manuscript and only provided financial support in the form of authors' salaries and/or research materials, please review your statements relating to the author contributions, and ensure you have specifically and accurately indicated the role(s) that these authors had in your study. You can update author roles in the Author Contributions section of the online submission form.

Please also include the following statement within your amended Funding Statement.

‘The funder provided support in the form of salaries for authors [insert relevant initials], but did not have any additional role in the study design, data collection and analysis, decision to publish, or preparation of the manuscript. The specific roles of these authors are articulated in the ‘author contributions’ section.’

If your commercial affiliation did play a role in your study, please state and explain this role within your updated Funding Statement.

2. Please also provide an updated Competing Interests Statement declaring this commercial affiliation along with any other relevant declarations relating to employment, consultancy, patents, products in development, or marketed products, etc.

Within your Competing Interests Statement, please confirm that this commercial affiliation does not alter your adherence to all PLOS ONE policies on sharing data and materials by including the following statement: ‘This does not alter our adherence to PLOS ONE policies on sharing data and materials.’ (as detailed online in our guide for authors <http://journals.plos.org/plosone/s/competing-interests>) . If this adherence statement is not accurate and there are restrictions on sharing of data and/or materials, please state these. Please note that we cannot proceed with consideration of your article until this information has been declared.

Please include both an updated Funding Statement and Competing Interests Statement in your cover letter. We will change the online submission form on your behalf.”

**Response**

We thank the editor for this comment. The funding and competing interest disclosures have now been clarified and included in the revised cover letter and manuscript.

**Funding statement:**
“National Natural Science Foundation of China (No. 81572522 and No. 81202011), Guangdong Innovation Platform Project (2012B090600021), Guangzhou 2024 Annual Special Project on Agricultural and Social Development Science and Technology (2024B03J1332, 2024B03J1249), and National Key R&D Program of China (2024YFA1307601, 2024YFA1307602, 2024YFA1307603).”

**Role of Funder statement:**
“The funders had no role in study design, data collection and analysis, decision to publish, or preparation of the manuscript.”

**Competing interests statement:**
“Ying Luo, Panning Wang, Shuhong Luo, Rochelle Wickramasekara and Ruo-Pan Huang are employees of and have a financial stake in RayBiotech, Inc. RayBiotech antibody arrays were used in this study. The company provided support in the form of salaries for these authors but did not have any additional role in study design, data collection and analysis, decision to publish, or preparation of the manuscript. This does not alter our adherence to PLOS ONE policies on sharing data and materials. The authors declare no additional competing interests.”

**Journal Requirement 6**

“6. In the online submission form you indicate that your data is not available for proprietary reasons and have provided a contact point for accessing this data. Please note that your current contact point is a co-author on this manuscript. According to our Data Policy, the contact point must not be an author on the manuscript and must be an institutional contact, ideally not an individual. Please revise your data statement to a non-author institutional point of contact, such as a data access or ethics committee, and send this to us via return email. Please also include contact information for the third party organization, and please include the full citation of where the data can be found.”

**Response**

We thank the editor for this comment. The Data Availability Statement has been revised to replace the co-author contact with a non-author institutional point of contact in accordance with the PLOS ONE Data Policy. Updated contact information and data access details are provided below.

Updated Data Availability Statement:

“The data supporting the findings of this study are available from the Office of Research Administration, The First Affiliated Hospital of Sun Yat-sen University, upon reasonable request. Contact person: Haifeng Lin, Research Officer. Email: [zsyykyc@mail.sysu.edu.cn](mailto:zsyykyc@mail.sysu.edu.cn); Phone: (086)020-87755766. Address: Office of Research Administration, The First Affiliated Hospital of Sun Yat-sen University, No. 58, Zhongshan 2nd Road, Guangzhou, Guangdong Province, China, 510080.”

**Journal Requirement 7**

“7. PLOS requires an ORCID iD for the corresponding author in Editorial Manager on papers submitted after December 6th, 2016. Please ensure that you have an ORCID iD and that it is validated in Editorial Manager. To do this, go to ‘Update my Information’ (in the upper left-hand corner of the main menu), and click on the Fetch/Validate link next to the ORCID field. This will take you to the ORCID site and allow you to create a new iD or authenticate a pre-existing iD in Editorial Manager.”

**Response**

We thank the editor for this comment. The corresponding author ORCID iD (<https://orcid.org/0000-0002-5884-8026>) has been validated and updated in Editorial Manager

**Journal Requirement 8**

“8. Please upload a new copy of Figures 1 to 6 as the detail is not clear. Please follow the link for more information: https://journals.plos.org/plosone/s/figures”

**Response**

We thank the editor for this comment. Figures 1–6 were regenerated and uploaded as revised high-resolution TIFF files in accordance with the PLOS ONE figure preparation guidelines to improve image clarity and readability. Supplementary figures were also updated accordingly.

**Journal Requirement 9**

“9. Please include captions for your Supporting Information files at the end of your manuscript, and update any in-text citations to match accordingly. Please see our Supporting Information guidelines for more information: http://journals.plos.org/plosone/s/supporting-information.”

**Response**

We thank the editor for this comment. Captions for all Supporting Information files were added to the end of the manuscript under a “Supporting information” section, and in-text citations were updated accordingly to match PLOS ONE formatting guidelines.

**Journal Requirement 10**

“10. If the reviewer comments include a recommendation to cite specific previously published works, please review and evaluate these publications to determine whether they are relevant and should be cited. There is no requirement to cite these works unless the editor has indicated otherwise.”

**Response**

We thank the editor for this comment. Reviewer comments and suggested revisions were evaluated and addressed where applicable in the revised manuscript.

# Reviewer 1.1

“In the cytokine array analysis methods, the authors report samples were homogenized in RayBio Lysis Buffer containing a protease inhibitor cocktail. It would be helpful to have the part numbers for both of these products, as they have for other materials.”

**Response**

We thank the reviewer for this comment. Additional methodological details were added to the cytokine array analysis section, including reagent catalog numbers, sample preparation procedures, chemiluminescent detection workflow, densitometric signal quantification, and normalization methods to improve reproducibility and methodological transparency.

Reviewer 1.2

“Generally, the methods are not in sufficient detail that the study could be replicated. More details should be added than “following manufacturer’s instructions”. In cases where algorithms or web servers were used, the authors should include settings/variables. For ELISA, the authors should report concentrations used for standard curves, as well as fits of the data to the standard curves. For KM Plotter, Affymetrix ID(s) for each gene should be reported (in addition to settings e.g. how high/low expression cutoff was selected and what the cutoffs were for each gene).”

**Response**

We thank the reviewer for this comment. The Methods section was expanded substantially to improve reproducibility and transparency. Additional methodological details were added for the cytokine array, ELISA, TCGA, Kaplan–Meier Plotter, and TIMER analyses, including data preprocessing, normalization procedures, statistical methods, differential expression criteria, and analysis parameters. For the cytokine array analysis, differential expression testing, Benjamini–Hochberg FDR correction, clustering methods, and pathway enrichment procedures are now explicitly described. For TIMER analyses, the database version, TCGA dataset, correlation method, tumor purity adjustment, significance criteria, and analyzed genes are now reported. Kaplan–Meier Plotter query parameters and gene symbol–based analyses have also been clarified in the revised Methods section.

Reviewer 1.3

“The cohorts should be better defined with standard and RCC context dependent epidemiological and demographic descriptors, such as age, gender, tobacco use, BMI, presence of hypertension, comorbidities, etc. Were all renal cancer patients chemotherapy naïve? If not, do the patients who received therapy or have distinct expression patterns from ones who did not? Or perhaps different therapies segregate into characteristic expression patterns. Overall, tumor vs paratumor vs normal is an oversimplification and the results of this study could be entirely driven by hidden variables.”

**Response**

We thank the reviewer for this important comment. We agree that clinical and demographic variables may contribute to heterogeneity in cytokine expression patterns and should be considered when interpreting the findings.

To address this concern, we revised the Patient Cohorts and Clinical Sample Collection section to provide additional clinicopathological information for the analyzed cohorts. The revised manuscript now clarifies that the tissue cohorts consisted predominantly of localized early-stage RCC (T1–T2) cases and were composed primarily of clear cell RCC tumors. Available clinicopathological characteristics, including age, sex, tumor size, T stage, and metastatic status, are now described in the Methods section, and Table 1 has been updated to include metastatic status.

We acknowledge that additional epidemiological and clinical variables, including smoking history, BMI, hypertension status, comorbidities, and detailed treatment history, were not available for all retrospective samples included in this study. Consequently, stratified analyses based on these variables could not be performed.

To further address this limitation, we expanded the Discussion section to acknowledge the molecular and immune heterogeneity of RCC and the possibility that unmeasured clinical variables may contribute to the cytokine expression patterns observed. We now emphasize that this study should be interpreted as an exploratory cytokine profiling analysis and that larger, clinically well-annotated cohorts will be required to further evaluate the influence of patient- and disease-specific variables on cytokine-associated signaling patterns in RCC.

Reviewer 1.4

“Figure 2 does not appear to have statistical comparisons. It should, which would also require multiple comparison corrections as there are more than 2 samples being compared per gene.”

**Response**

We thank the reviewer for this comment. Statistical comparisons and significance annotations have been added to Figure 2. In addition, the figure layout was revised to improve clarity, with the representative antibody array images now presented as Panel A and the corresponding quantified signal intensity data presented as Panel B. The figure legend has also been expanded to provide additional detail regarding the displayed cytokines and sample groups. The Methods section was revised to clarify the statistical framework used for cytokine array analysis, including moderated linear modeling, multi-group comparisons, pairwise testing, and Benjamini–Hochberg FDR correction for multiple testing.

Reviewer 1.5

“In general, the figure legends need improved descriptions that allow for essentially full interpretability without requiring digging into the text or external material.”

**Response**

We thank the reviewer for this comment. All figure legends were revised to improve standalone interpretability and reduce the need to reference the main text. The revised legends now provide additional detail regarding sample groups, experimental methods, statistical annotations, panel organization, and the principal findings illustrated in each figure. Supplemental figure legends were also expanded to improve clarity and reproducibility.

Reviewer 1.6

“Typically, there is not a strong correlation between gene and protein expression. This is especially true for secreted proteins, particularly cytokines. There are many regulatory mechanisms between RNA expression and functional proteins being released and used. Cytokines also tend to have short half-lives, are active distal to their production site, and are by nature downstream of a more fundamental regulator. These facts combine to explain why there is poor correlation between results in the manuscript (compare patterns for each gene across figures). Often the antibody array (Fig 2), ELISA (Fig 3), and TCGA RNA (Fig S1) disagree with each other. It is difficult to imagine prognostic and mechanistic relevance of these transient signaling molecules taken at a single timepoint with such wide within-group distributions and lack of agreement between different assays. In fact, the 5th discussion paragraph states “these findings highlight the potential inconsistency between cytokine protein levels and mRNA expression when analyzing renal tumor samples”. This is of course true but extends more broadly than renal tumor samples. It’s also why we need to cautiously assess the significance of protein/RNA findings that oppose each other.”

**Response**

We thank the reviewer for this important comment. We agree that cytokine protein abundance, circulating cytokine levels, and transcriptomic expression are not expected to demonstrate complete concordance, particularly for secreted cytokines that are subject to complex regulation at multiple biological levels. We also agree that transcriptomic findings should not be interpreted as direct validation of protein-level observations.

To address this concern, we substantially revised the Results and Discussion sections to present the TCGA analyses as complementary transcriptomic contextualization rather than validation of the antibody array or ELISA findings. For example, in the Results section we now state that: “these transcriptomic analyses were interpreted primarily as complementary contextual analyses rather than direct validation of the protein-level findings” (Results, TCGA transcriptomic analyses section).

We also expanded the Discussion to explicitly acknowledge the biological factors that may contribute to discordance between protein- and RNA-based measurements. Specifically, we now state that: “These findings highlight the complex relationship between cytokine protein abundance and transcriptomic expression within the RCC tumor microenvironment” and that “cytokine expression patterns may vary substantially according to tumor stage, immune cell composition, local inflammatory conditions, secretion dynamics, and post-transcriptional regulatory mechanisms, potentially contributing to discordant findings across tissue protein- and RNA-based analyses” (Discussion, IL-2/IL-15 section).

In addition, we expanded the study limitations to acknowledge the incomplete concordance observed across tissue protein expression, circulating cytokine measurements, and transcriptomic datasets. The revised manuscript now states: “Incomplete concordance was observed across tissue protein expression, circulating cytokine measurements, and transcriptomic datasets,” and further notes that such variability “is consistent with the multi-layered regulation of cytokine expression and signaling” (Discussion, Limitations section).

Overall, these revisions provide a more cautious interpretation of the multi-platform findings and better acknowledge the biological complexity and limitations associated with relating cytokine protein measurements, circulating biomarkers, and transcriptomic expression data.

Reviewer 1.7

“In the section entitled “several upregulated cytokines correlate with immune cells in early stage RCC” the type of correlation (e.g. Pearson, Spearman, etc) is not stated, nor is this in the methods section. I also suggest pruning Figure 5 to include only the most significant ones to draw attention to them, rather than losing them in the large mix of mostly insignificant genes; the full set can be a supplemental figure. This will improve interpretability of the figure. It should also be stated more clearly that it is the gene level data from TCGA, which is not necessarily the same as the cytokine (protein) level itself.”

**Response**

We revised this section and corresponding figure legend to clarify that these analyses were performed using TCGA-KIRC transcriptomic data and TIMER-based immune infiltration estimates rather than direct cytokine protein measurements. The Results text was also revised to improve interpretation of these findings and avoid overstatement.

In addition, Figure 5 was simplified to focus on the strongest cytokine–immune cell associations (LPS/CD14, AXL, CXCL9, CCL4, and CCL5) in the main figure, while additional correlation analyses were moved to S3 Fig to improve clarity and interpretability.

The statistical correlation method and TIMER analysis parameters have now been explicitly described in the Methods section, including the use of Spearman correlation analysis with tumor purity adjustment.

Reviewer 1.8

“Overall, the manuscript should improve transitions between results sections and the results themselves, which are little more than lists.”

**Response**

We thank the reviewer for this helpful suggestion. To improve readability and narrative flow, the Results section was extensively revised to reduce list-like presentation of findings and strengthen transitions between major analyses. Rather than presenting sequential observations as isolated results, we added contextual statements that explain how each analysis relates to the overall study objective and how subsequent analyses build upon preceding findings.

For example, following the cytokine array analysis, we added text stating that “these findings suggest that early-stage RCC is associated with coordinated alterations in angiogenic, inflammatory, and immune regulatory signaling pathways,” thereby providing a biological framework for the differentially expressed cytokines before introducing downstream validation studies. Similarly, before presenting the TCGA analyses, we added text explaining that these analyses were performed to provide “complementary transcriptomic context for the observed protein-level alterations” rather than introducing the dataset abruptly.

Additional transitions were incorporated throughout the Results section to improve continuity between the antibody array profiling, ELISA validation, TCGA transcriptomic analyses, and TIMER immune infiltration analyses. These examples are representative of broader revisions made throughout the Results section to improve organization, interpretation, and overall readability.

Reviewer 1.9

“In the discussion section 2nd paragraph, there are proteins stated that are not cytokines, but more importantly this study did not validate through their assays. The discussion should clearly differentiate and cite previous findings in the literature from specific cytokines validated through the assays in this study. It should also differentiate cytokines from the up or downstream proteins that regulate or are regulated by them.”

**Response**

We thank the reviewer for this helpful comment. We agree that the proteins identified in the antibody array represent a diverse group of cytokines, chemokines, growth factors, receptors, adhesion molecules, and other immune regulatory proteins, and that these categories should not be discussed interchangeably. We also agree that findings identified in the discovery array should be clearly distinguished from those that underwent additional validation analyses.

To address this concern, the Discussion was substantially revised to replace broad references to “cytokines” with more precise descriptions such as “cytokine-associated signaling patterns,” “angiogenesis-associated signaling molecules,” “inflammatory chemokines,” and “immune regulatory signaling molecules,” where appropriate. For example, the revised Discussion now states that “our exploratory cytokine profiling analyses identified altered expression patterns involving angiogenesis-associated, inflammatory, and immune regulatory signaling molecules in early-stage RCC tissues” rather than grouping all identified proteins under a single cytokine category.

In addition, the revised Discussion now distinguishes observations derived from the initial antibody array screen from findings that underwent subsequent evaluation in independent tissue, serum, or urine cohorts. Specifically, we added the statement: “Notably, selected findings from the discovery cohort, including alterations in Angiopoietin-2, CCL4, CXCL9, HGF, and ErbB3, were further evaluated in independent tissue, serum, or urine validation analyses.” This clarification avoids presenting all detected proteins with equivalent levels of validation.

We also revised the Discussion to more clearly differentiate cytokines and chemokines from growth factors, receptors, and downstream signaling-associated proteins when discussing their potential biological relevance. These changes were made throughout the Discussion to improve biological accuracy, distinguish validated findings from discovery-stage observations, and avoid overinterpretation of the screening results.

Reviewer 1.10

“The third and fourth paragraphs of discussion highlight that the variability in the measurements are nearly as large as the measurements themselves. While there may be statistically significant differences between tumor and normal samples, it is possible there are subsets of tumor samples which drive this, while other subsets rely on a different mechanism. Some sort of clustering or similar approach could be taken to segregate the tumor samples into molecularly defined subtypes. These might be related to treatment vs naïve, or one of the epidemiological/demographic features.”

**Response**

We thank the reviewer for this insightful comment. We agree that RCC is a heterogeneous disease and that distinct molecular subgroups may contribute to the variability observed in cytokine expression patterns.

The primary objective of this study was to identify cytokine alterations associated with RCC through exploratory protein profiling and subsequent validation analyses. Due to the relatively limited cohort size and incomplete availability of detailed clinical variables, robust subgroup or clustering analyses could not be performed reliably.

To address this concern, we expanded the Discussion section to acknowledge the biological and clinical heterogeneity of RCC and the possibility that the observed cytokine patterns may reflect multiple underlying disease subtypes. We now note that larger, clinically annotated cohorts will be required to evaluate molecular subgroups and determine whether distinct cytokine signatures are associated with specific clinicopathological characteristics or disease states.

Reviewer 1.11

“The discussion and conclusion should be more carefully worded given the inherent limitations of using single timepoint sampling on very transient signaling proteins, lack of agreement with the protein and RNA, etc.”

**Response**

We thank the reviewer for this important comment and agree that cytokine measurements represent dynamic biological processes that should be interpreted cautiously. To address this concern, we substantially revised the Discussion and Conclusion sections to adopt a more cautious interpretation of the findings and expanded the limitations section.

Specifically, we now acknowledge the observed discordance across analytical platforms, stating that “incomplete concordance was observed across tissue protein expression, circulating cytokine measurements, and transcriptomic datasets.” We also added a limitation noting that “the present analyses were based on samples collected at a single clinical time point, limiting assessment of temporal changes in cytokine expression and tumor microenvironment remodeling during RCC progression.” Finally, we note that “additional mechanistic and longitudinal studies will be required to clarify the functional significance and temporal evolution of these signaling networks during RCC progression.”

These revisions provide a more balanced interpretation of the findings and more clearly acknowledge the limitations inherent to exploratory cytokine profiling studies.

Reviewer #2

Reviewer 2.1

“The discovery cohort includes 20 paired samples, and validation cohorts are also relatively small. Given that 174 cytokines were profiled, this raises concerns about statistical robustness.

Please provide a justification for sample size or power calculation.
Expand discussion of this limitation.”

**Response**

We thank the reviewer for this comment. We agree that the relatively small discovery and validation cohorts represent an important limitation of the study. To address this concern, we expanded the limitations section of the Discussion to more explicitly acknowledge the exploratory nature of the study, the relatively small cohort sizes, and the need for validation in larger independent patient cohorts. Specifically, the revised manuscript now states that the study was “designed as an exploratory cytokine profiling analysis” and that “larger independent cohorts will be necessary to further evaluate the reproducibility and generalizability of the cytokine-associated signaling patterns identified in this study.”

Reviewer 2.2

“You identified 34 differentially expressed cytokines, but no correction for multiple comparisons appears to have been applied.

Please apply false discovery rate (FDR) correction or similar methods.
Reassess which cytokines remain significant after correction.”

**Response**

Differential expression analysis of the cytokine array data was performed using the limma package in R. FDR correction was applied using the Benjamini–Hochberg procedure, and cytokines with FDR < 0.05 were considered significantly differentially expressed. The Methods section has been revised to explicitly describe the statistical procedures used for differential expression analysis and multiple-testing correction. The final set of 34 cytokines was selected from the significantly differentially expressed cytokines based on statistical significance, fold change, and biological relevance to RCC-associated signaling pathways.

Reviewer 2.3

“The manuscript lacks sufficient detail regarding array normalization methods, handling of batch effects, and quality control procedures. This limits reproducibility.

Please clearly describe data preprocessing steps.
Consider including quality control plots or supplementary figures.”

**Response**

We expanded the cytokine array Methods section to provide additional detail regarding array preprocessing, normalization, and quality control procedures. Specifically, we clarified that duplicate spot intensities were averaged following background subtraction, signal intensities were normalized to internal positive control spots, and duplicate spots and internal controls were used to assess signal consistency across membranes. Additional methodological detail regarding densitometric signal quantification, clustering analysis, and pathway enrichment workflows was also added to improve reproducibility and interpretability of the array analyses.

Reviewer 2.4

“You report inconsistencies between protein (ELISA) and mRNA (TCGA) levels for several cytokines such as IL-2 and IL-15.

This is an important observation but is not sufficiently explored.
Please provide a more detailed discussion addressing potential biological explanations such as post-transcriptional regulation or secretion dynamics.”

**Response**

We thank the reviewer for this important observation. To address this concern, we substantially expanded the Discussion of discordant protein- and transcript-level findings, particularly for IL-2 and IL-15. The revised Discussion now states that “these findings highlight the complex relationship between cytokine protein abundance and transcriptomic expression within the RCC tumor microenvironment” and further notes that cytokine expression patterns may vary according to “tumor stage, immune cell composition, local inflammatory conditions, secretion dynamics, and post-transcriptional regulatory mechanisms, potentially contributing to discordant findings across tissue protein- and RNA-based analyses.” We also expanded the limitations section to acknowledge the incomplete concordance observed across tissue protein, circulating cytokine, and transcriptomic datasets and to emphasize the need for cautious interpretation of these multi-platform findings.

Reviewer 2.5

“The claim that urinary ErbB3 may serve as a non-invasive biomarker is interesting but currently not well supported.

The validation cohort is small.
Please tone down the conclusion and present this as a preliminary finding.”

**Response**

We thank the reviewer for this comment and agree that the urinary ErbB3 findings should be interpreted cautiously given the relatively small validation cohort. To address this concern, we substantially toned down the discussion of ErbB3 as a potential biomarker and now present these observations as preliminary findings requiring further validation. Specifically, the revised Discussion states that “larger validation studies will be required to determine the potential biological or clinical relevance of these observations” rather than suggesting clinical utility. We also removed stronger biomarker-oriented language from the summary and conclusion sections to better reflect the exploratory nature of the study.

Reviewer 2.6

“The study is primarily descriptive and does not include mechanistic experiments.

Please acknowledge this limitation more explicitly and avoid causal interpretations.”

**Response**

We thank the reviewer for this comment and agree that the present study is primarily exploratory and descriptive in nature. To address this concern, we revised the manuscript to more clearly frame the work as an exploratory characterization of cytokine-associated alterations in early-stage RCC and substantially revised the Discussion and Conclusion sections to avoid causal interpretations. In addition, the limitations section now explicitly acknowledges that “additional mechanistic and longitudinal studies will be required to clarify the functional significance and temporal evolution of these signaling networks during RCC progression.” These revisions are intended to ensure that the findings are presented as associative observations rather than direct evidence of causality.

Reviewer 2.7

“There is limited discussion of patient heterogeneity.

Please clarify whether variables such as age, sex, tumor subtype, or grade were considered.
Include additional clinical context if available.”

**Response**

We thank the reviewer for this comment. Additional clinical context has been incorporated into the revised manuscript. The Patient Cohorts and Clinical Sample Collection section now clarifies that the tissue cohorts consisted predominantly of localized early-stage RCC (T1–T2) cases and were composed primarily of clear cell RCC tumors. Available clinicopathological characteristics, including age, sex, tumor size, T stage, and metastatic status, are described in the Methods section, and Table 1 has been updated to include metastatic status.

We acknowledge that additional clinical variables, including detailed comorbidity information and other epidemiological characteristics, were not available for all retrospective samples included in this study. The Discussion section has been expanded to recognize patient heterogeneity and the potential contribution of unmeasured clinical variables to the observed cytokine expression patterns as a limitation of the study.

Reviewer 2.8

“Use of t-tests for multiple comparisons may not be appropriate.

Please clarify whether assumptions of normality were tested.
Consider non-parametric approaches where appropriate.”

**Response**

We thank the reviewer for this comment. The Statistical Analysis section has been revised to provide additional detail regarding the statistical methods used throughout the study. Comparisons between two groups were performed using Student's t-test or paired t-test as appropriate, while comparisons involving more than two groups were analyzed using one-way ANOVA followed by post hoc multiple-comparison testing. In addition, the cytokine array analysis was revised to explicitly describe the limma-based statistical framework and Benjamini–Hochberg FDR correction used for differential expression analysis.

Reviewer 2.9

“Some grammatical issues and repetitive phrasing are present. A careful language edit would improve readability.

Figures are informative but need improvement. Units should be standardized, for example pg/ml should be used consistently. Figure legends should also be more descriptive.

Terminology should be consistent throughout the manuscript, particularly terms such as para-tumor and para-cancerous tissue.”

**Response**

We thank the reviewer for these helpful suggestions. The manuscript has undergone extensive language editing to improve grammar, readability, and overall flow. Figure legends were substantially expanded to provide clearer descriptions of the experimental design, sample groups, analytical approaches, and data interpretation. In addition, terminology was reviewed and standardized throughout the manuscript, including consistent descriptions of tissue sample types. Units and figure labeling were also reviewed for consistency across the revised manuscript and figures.
