# Supplementary material for: Tumor specific cytokine pattern explored in renal cell carcinoma
Source: PLoS One. 2026 Sep 29;21(9):e0358533. doi: 10.1371/journal.pone.0358533 (PMC13623153; doi:10.1371/journal.pone.0358533)
Supplement: Supplementary file 2 [file pone.0358533.s007.docx]

# PLOS ONE Revision Response Document

**Manuscript ID:** PONE-D-26-01255
**Title:** *Tumor Specific Cytokine Pattern Explored in Renal Cell Carcinoma*

**Reviewer #1**

**Comment 1**

The authors appear to have addressed most of the peer review concerns. I appreciate the significant efforts to include method and cohort details (and state that certain data were not available). The statistical approaches seem reasonable given the study limitations.

**Response:** We thank the reviewer for the positive assessment and appreciate the constructive feedback provided throughout the review process.

**Comment 2a**

Overall, the main points that still remain are the Affymetrix IDs and cutoff values for each gene. These matter for reproducibility because KM Plotter can map one gene to multiple different probe sets and the cutoff value changes periodically even when automatic settings are used.

**Response:** We thank the reviewer for this comment. Upon further review of the Kaplan–Meier Plotter analysis, we recognized that our previous description did not clearly specify the type of gene-expression dataset used. The survival analyses were performed using the **Pan-cancer RNA-seq dataset** for TCGA-KIRC, rather than an Affymetrix microarray dataset. Affymetrix probe-set IDs, which identify the specific microarray probes used to measure gene expression, are therefore not applicable to these RNA-seq analyses. We have clarified this distinction in the revised Methods and S2 Fig legend.

To further improve reproducibility, we have also added the exact expression cutoff used to stratify patients into high- and low-expression groups for each gene: ANGPT2, 3,147; IL2, 2; IL15, 250; CXCL9, 2,340; CCL4, 382; HGF, 521; ERBB3, 3,076; FLT3LG, 69; and IGFBP2, 708. These cutoffs were selected using the Kaplan–Meier Plotter “auto select best cutoff” setting, as described in the revised Methods.

**Comment 2b**

The ELISA standard curve concentrations and the curve fit statistics matter for reproducibility, but also to judge how well the sample concentrations could be interpolated from the assay based on both detection range and good fit of the standard curve to the regression model.

**Response:**

Thank you for this comment. We have now provided the standard concentration series used for each ELISA, together with the curve-fitting model and corresponding R² values, in a new Supporting Information table (S1 Table). Standard curves were fitted using a four-parameter logistic (4PL) regression model, with R² values ranging from 0.9938 to 1.0000. The ELISA Methods section has also been revised to specify the curve-fitting model and direct readers to S1 Table. These additions provide the standard concentration ranges and goodness-of-fit information for the ELISA assays and improve reproducibility of the reported measurements.

**Comment 3**

The authors included much more detail into the statistical methods, stating which tests and corrections were used. However, it still isn’t clear that normality was checked (e.g. Shapiro-Wilk) and that the data were normally distributed.

**Response:** We thank the reviewer for this comment. To directly address this concern, we performed Shapiro–Wilk normality testing for the relevant datasets and re-evaluated the statistical comparisons using distribution-appropriate methods. The Statistical Analysis section has been revised to explicitly describe the normality assessment and selection of parametric or non-parametric tests.

Overall, the revised analyses were consistent with the previously reported expression patterns, with the direction of cytokine changes remaining unchanged. Most statistical conclusions were also retained, with only a few significance annotations requiring revision. In Fig. 2, IL-2 remained lower in tumor tissues but no longer reached statistical significance; this has now been explicitly stated in the Results and figure legend. Notably, reduced IL-2 expression remained significant in the independent ELISA validation cohort (Fig. 3). In the ELISA analyses, PDGF-AA in tissue (Fig. 3) and MIP-1β/CCL4 in urine (Fig. 6) reached statistical significance following the revised analyses.

Figs. 2, 3, and 6 and the corresponding Results, figure legends, and Statistical Analysis section have been updated accordingly.

**Comment 4**

If those last things are addressed, the study will be publishable.

**Response:** We thank the reviewer for this assessment. The remaining comments have been addressed as described below.
